# The evolution of a healthcare interpreting service mapped against the bilingual health communication model: a historical qualitative case study

**DOI:** 10.1186/s40985-020-00123-8

**Published:** 2020-08-04

**Authors:** Alexander Bischoff

**Affiliations:** 1 School of Health Sciences, University of Applied Sciences and Arts Western Switzerland, Route des Cliniques 15, CH-1700 Fribourg, Switzerland; 2 Institute of Global Health, Faculty of Medicine, University of Geneva, 24 rue du Général-Dufour, 1211 Genève 4, Switzerland

**Keywords:** Healthcare interpreting, Migrant health, Refugees, Asylum seekers, Language barriers, Diversity, Equity

## Abstract

**Background:**

Twenty-five years ago, the need for health care interpreting in Switzerland increased due to the sharp influx of asylum seekers from war zones and countries of political unrest. Due to complex health needs, there was a need to move away from using volunteers as interpreters towards qualified interpreter services.

**Methods:**

A historical qualitative case study design was used to describe the evolution of the language assistance programmes at Geneva University Hospitals, between 1992 and 2017. The aim was to map the evolution of the interpreter services against the Bilingual Health Communication Model with the constructs—Communicative Goals, Individual Agency, System Norms and Quality and Equality of Care—while identifying key factors to optimise interpreter service and patient care.

**Results and discussion:**

Five phases were identified during the 25 years of service evolution studied: (1) Service initiation—the interpreter services were first used in a small service that cared for refugees and asylum seekers. (2) Growth and formalisation—due to the arrival of high numbers of Albanian-speaking asylum seekers, Albanian-speaking interpreters were provided to all departments of the Geneva University Hospitals. This helped roll out the use of interpreters among doctors and nurses. (3) Ensuring quality—the care for all patients, whether foreign-language speaking or not, became an issue and led to research into the quality of patient-provider communication. (4) Institutionalisation—this phase dealt with challenges including the lack of interpreter financing regulation and the clarification of interpreter roles. (5) Equity—healthcare interpreter services were put in an overall framework of equity standards. The Bilingual Health Communication Model was applied and showed that some items were not implemented: clear shifts (i) towards a culturally sensitive focus, (ii) towards community interpreting, (iii) towards triadic communication, (iv) towards spelling out the right to have an interpreter and (v) towards the involvement of insurance companies. Finally, the inclusion of healthcare interpreting as an essential ingredient in healthcare provision, including chronic disease management, is incomplete or missing.

**Conclusions:**

Healthcare interpreting at Geneva University Hospitals has evolved from a ‘muddling-through’ approach towards an institutional approach by addressing quality of care, by focussing on the mental health of asylum seekers, training of both interpreters and users of interpreters and institutional policy based on equity.

## Background

Cities in the Western world have been facing unprecedented diversity over the last decades. Many cities have become ‘majority–minority cities’ ([1]:57), meaning that they consist of only minorities. For example, Amsterdam and Brussels recently became such majority–minority cities; London and Paris will be soon. This trend has been described as ‘the diversity turn’ [1]. Often diversity has been seen only in terms of ethnicity. Other variables of diversity include different immigration statuses, respective entitlements and rights, divergent labour market experiences, demographic practices, patterns of spatial distribution and, importantly, multilingualism. Vertovec considering all these facets of diversity proposed the term ‘super-diversity’ ([2]:1025).

Although the growth of multilingualism has been recognised by researchers and policy-makers, responses to overcome the difficulties this presents have rarely been adequate ([2]:1032). Quite strikingly, language assistance programmes do not feature prominently in policy development on diversity, including the specific mention of interpreters. This is all the more unexpected since health research provides ample evidence that language barriers do lead, if left unaddressed, to poor patient outcomes: for instance, patients facing language barriers are more likely to experience adverse events in hospitals [3], more likely to be non-adherent to self-monitoring prescriptions [4], more likely to be denied psychological follow-up [5], more likely to be dissatisfied with care [6], more likely to suffer from medical errors [7] and a poor therapeutic relationship [8], more likely to experience longer length of stay (LOS) [9] and more likely to be denied treatment options [10].

There is also an increasing body of evidence that the use of healthcare interpreters reduces healthcare inequalities for foreign language patients [11–15]. For example, the use of interpreters correlated with a lower number of visits to an outpatient facility and with more targeted care; moreover the escalation of long-term costs, especially in chronically ill patients, was prevented [16].

Healthcare interpreting has been emerging in Western cities over the last 25 years or so.

Globalisation, migration, arrival of refugees from all over the world and overall increasing mobility triggered the emergence of language assistance programmes in these contexts. Interestingly, an exception is Australia: It is the country with the best developed system of interpreter services, including certification. Since WWII, when Australia actively encouraged immigration, language access had to be provided for the immigrants most of whom had not an English-speaking background [17–19].

Healthcare interpreters are considered to be community interpreters. ‘In the most general sense, community interpreting refers to interpreting in institutional settings of a given society in which public service providers and individual clients do not speak the same language’ ([20]:126). Other terms used in the literature and synonymous to community interpreting include ‘public service interpreting’ or ‘liaison interpreting’.

In this study, the term healthcare interpreting is used throughout, although 30 years ago, medical interpreting was more commonly used, as if the use of interpreters was only relevant for physicians or in the world of medicine. Also, the term ‘translator’ was used for many years, although wrongly, because translation refers to written and not oral work.

Today, healthcare interpreting is coming of age. It has taken a long time to develop from muddling-through approaches towards institutionalised forms of interpreter services that are part of today’s health services in countries characterised by the diversity turn.

The reason for this slow evolution might be a general lack of conceptual frameworks of healthcare interpreting. Recently, Hsieh came up with such a conceptual framework that she labelled Bilingual Health Care (BHC) Model [21]. Hsieh conceptualises health care interpreting as a goal-oriented communicative activity that involves active participation from all the participants—not only provider, patient and interpreter, but also other participants (family members, for example). This model delineates ‘how participants’ diversity, agenda and agency in the medical encounter shape their role performances and expectations (for self and others) during the communicative process’ ([21]:3). In addition, institutional structures and cultures, as well as medical ethics and values, all contribute to shaping participants’ behaviours, social norms and communicative strategies. The four constructs of the BHC Model are Communicative Goals, Individual Agency, System Norms, and Quality and Equality of Care (QEC). Concentrically organised, with communicative goals as the inner circle and QEC as the outer one, ‘[a]ll these constructs are applicable to all participants in the medical encounter. However, individuals’ understanding, assessment and skill level for these constructs may differ. In addition, individuals may hold competing (and potentially conflicting) understandings of these constructs, resulting in tensions and challenges in interpreter-mediated medical encounters’ ([21]:137).

So far, and to my knowledge, little if any attempts have been made to look at the evolution of healthcare interpreting in a historical perspective.

In this study, I examine the emergence of an interpreter service and its institutionalisation. The historical case study aims (i) to describe the evolution of the interpreter services in GUH, (ii) interpret the evolution against the BHC model and (iii) identify key factors to optimise interpreter service and patient care.

## Methods

### Research design

The methodological approach we use is a historical qualitative case study design [22]. This approach is useful, because case studies have a long history in qualitative research, ‘draw on the ability of the qualitative researcher to extract depth and meaning in context’, and ‘play an important role in programme evaluation’ ([23]:5). Flyvbjerg notes that ‘a discipline without a large number of thoroughly executed case studies is a discipline without systematic production of exemplars, and a discipline without exemplars is an ineffective one’ ([23]:6). Amenta, in ‘Making the Most of an Historical Case Study’, supports research that ‘tends to seek less general claims rooted in more in-depth knowledge’ ([24]:353).

Therefore, I have focused this study on the canton of Geneva of Switzerland and more precisely on Geneva University Hospitals (GUH) to identify factors and influences that paved the way incrementally from muddling-through (‘grass-root’) interpreting to institutionalisation.

### Sampling

In my historical case study, I aimed to analyse the factors promoting or hindering the emergence of interpreter services at GUH. I included materials from the last 25 years. By using convenience sampling, I obtained data consisting of (a) publications (peer-reviewed and not-peer-reviewed, data-based or policy papers) and (b) reports dealing with interpreter services, books, book chapters and policy documents on migration and health, asylum seekers and refugees, assimilation and integration, health(care) disparities and access to equitable healthcare. These materials were produced at cantonal and national level (Federal Office of Public Health, Federal Office for Migration, Federal Office of Statistics, Swiss Forum for Migration and Population Studies), as well as data gathered during the Swiss National Funding call—‘The interdisciplinary National Research Programme, Social Integration and Social Exclusion’). This research programme analysed the emergence, modes of functioning and social consequences of processes of social integration and social exclusion in Swiss society. The author was the principal investigator of the project called ‘Does intercultural mediation contribute to inclusion? Comparing policies and practices in the sectors of health, education, social and legal services’ [25, 26]. Table 1 displays all sources included in this study.

**Table 1:** Data collected that contain information on healthcare interpreting in Geneva

Period	Publications (these include journal publications both peer-reviewed (marked*) and non-peer reviewed)	Other documents (these include reports, book chapters, books)
1992–1995 (‘initiation’)	On health care for asylum seekers Les soins aux requérants d’asile: une médecine à part? [27] * Medical Screening of Asylum Seekers in Switzerland [28] Afflux de réfugiés en Suisse: quels problèmes de santé? [29] Torture et violence organisée: leurs conséquences sur les requérants d’asile et les réfugiés [30] * The health of asylum seekers: from communicable disease screening to post-traumatic disorders. [31] * Identification of victims of violence and torture: the practitioner’s role [32] La santé des requérants d'asile: des parasites au stress post-traumatique [33] L’examen sanitaire de frontière appliqué aux requérants d’asile [34] Die Prävalenz von HIV-Infektionen und sexuell übertragbaren Krankheiten in der Schweiz. Bulletin des Bundesamtes für Gesundheit. 1993 [35] Tuberkulose in der Schweiz 1994 [36] Grenzsanitarische Untersuchung bei Asylbewerbern [37] Tuberculose multirésistante en Suisse: surveillance globale de la résistance aux médicaments [38] On the migratory context in Europe, Switzerland and Geneva Health Policies for Immigrant Populations in the 1990s. A Comparative Study in Seven Receiving Countries [39] Migration and international health policies [40] * No real progress towards equity: the health of migrants and ethnic minorities on the eve of the year 2000 [41] * Early texts on intercultural communication, relevant for the context of Geneva: L’interprète: traducteur, médiateur culturel ou co-thérapeute [42] Interkulturelle Kommunikation im Gesundheitsbereich [43] Eine Übersetzerin der Hausarztpraxis, Kommunikation mit fremdsprachigen Patienten [44]	Geneva-context related information Soigner les migrants: une affaire de spécialistes ou de généralistes? [45] Examen sanitaire de frontière des requérants d’asile à Genève. Genève: OFSP [46] Country reports in Migration and Health in Europe. [47] Migration and Health in Switzerland [48] Die Sprachenlandschaft Schweiz [49] Santé des migrants: l’interprétariat médical, aspect incontournable de la prise en charge [50] Asylum seekers, refugees and health in Switzerland. [51] Asylum seekers, refugees and health in the nineties [51] Analytic review of migration and health and as it affects European Community countries [52]
1995–1999 (‘growth’)	On facilitating communication with and healthcare access of asylum seekers, Geneva and Switzerland Asylum seekers and refugees in the medical polyclinic: a comparison between the Basel, Bern and Geneva polyclinics [53] * Asylsuchende und Flüchtlinge in der Notfallstation [54] * Asylsuchende und Flüchtlinge in der hausärztlichen Praxis: Probleme und Entwicklungsmöglichkeiten [55] * On facilitating communication with migrant patients, Geneva and Switzerland Die Arzt-Patienten Interaktion aus der Sicht von MigrantInnen: Vorschläge für die ärztliche Praxis [56] On introducing interpreter services in healthcare, Geneva and Switzerland The importance of interpreters to insure quality of care for migrants [57] * Language difficulties in an outpatient clinic in Switzerland [58] * Addressing language barriers to health care, a survey of medical services in Switzerland [59] * Medical interpreters have feelings too [60] * Wenn PatientInnen und Behandelnde nicht dieselbe Sprache sprechen... - Konzepte zur Übersetzerpraxis [61] * Interpreting in Swiss hospitals [62] * Sprachbarrieren und Kommunikation in einer medizinischen Poliklinik [63] Von einer Sprache zur anderen: Kommunikation mit fremdsprachigen Patienten in einer medizinischen Poliklinik [64] On specific settings where interpreters are needed Use of interpreters in Switzerland's psychiatric services [65] * Migrationspezifische Aspekte in einem psychotherapeutischen Prozess [66] Barrières linguistiques et communication dans une policlinique de médecine [67] On Health and migration in Europe (including information related to Geneva) Asylum seekers in Europe: entitlements, health status, and human rights issues [68] * Migration and health in the European Union [69]	Geneva context-related information Übersetzerinnen im Gesundheitsbereich: das medizinische Anamnesegespräch im Migrationskontext [70] Übersetzung und kulturelle Mediation im Gesundheitssystem [71] Migration und Gesundheit: interdisziplinäre Perspektiven und Stand der psychosozialen Forschung [72] The community interpreter’s task: self-perception and provider views [73] Interpreting & translating in Australia: current issues and international comparisons. [18] Knackpunkte im dolmetschervermittelten Gespräch [74] Communication interculturelle et accès aux soins, le défi du multilinguisme dans le contexte médical [75] Manuel du questionnaire sur l’état de santé des requérants d’asile [76]
1999–2003 (‘quality’)	Research on improving communication with migrants, Geneva: Improving communication between physicians and patients who speak a foreign language [77] * Measuring quality and patient satisfaction in healthcare communication with foreign-language speakers [78] * On the arrival of large numbers of Kosova refugees and the implications for interpreter provision, Geneva Screening of mental disorders in asylum-seekers from Kosovo [79] * Dolmetscher für Kosova-Flüchtlinge, Kurzbericht aus Genf [80] Gesund werden erfordert verstanden werden. Gute Erfahrungen mit professionellen Dolmetscherdiensten [81] On striving towards qualified interpreter services, Geneva and Switzerland Vermitteln zwischen Sprachen und Kulturen. Ausbildungs- und Qualitätsstandards sind vorgesehen [82] Ausgeschlossen durch die fremde Sprache [83] Sprachverwirrung im Spital [84] Research into specific settings of bilingual health communication, Geneva Language barriers between nurses and asylum seekers: their impact on symptom reporting and referral rates [5] * Interpreter-mediated diabetes consultations: a qualitative analysis of physician communication practices [85] *	Geneva context-related information Migration et santé - Stratégie de la Confédération pour les années 2002 à 2006 [86] Caring for Migrant and Minority Patients in European Hospitals: A Review of Effective Interventions [87] Avis de la Commission d’Ethique Clinique sur l’interprétariat communautaire, quoted in [88] Dolmetschervermittelte Gespräche im Spital: Sinnvolle Professionalisierung [89] ‘Hätten Sie jemanden zur Hand, der übersetzen kann?’ Interkulturelles Übersetzen und Vermitteln im Gesundheitsbereich [90] Verloren in der Übersetzung [91] Der Mediator als Dolmetscher - der Dolmetscher als Mediator [92] Les barrières langagières dans les relations de soin [93] Fremde Sprachen im Spital [94] ‘Was sagt mir das Transkript?’ - Dokumentation einer Plenumsdiskussion [95] Analysing interpreted doctor-patient communication form the perspectives of linguistics, interpreting studies and health sciences [96] Macht Migration krank? Eine von Migrantinnen und Migranten [97] Video Trialogue - Dolmetschen im Gesundheitswesen [98]
2004–2010 (‘institutio-nalisation’)	On access to institutionalised interpreter services, Geneva, Switzerland Access to Healthcare Interpreter Services: Where Are We and Where Do We Need to Go? [99] * Communicating with foreign language-speaking patients: is access to professional interpreters enough? [100] * On costs of interpreter provision What do language barriers cost? An exploratory study among asylum seekers in Switzerland [16] * Do Asylum Seekers Consume More Health Care Resources? Some Evidence from Switzerland [101] * The cost of war and the cost of health care - an epidemiological study of asylum seekers [102] * On the role of gender in bilingual health communication Doctor-patient gender concordance and patient satisfaction in interpreter-mediated consultations: an exploratory study [103] * Der Dialog zu Dritt: PatientInnen, DolmetscherInnen und Gesundheitsfachleute in der Universitäts-Frauenklinik in Basel [104] * Reproductive health care for asylum-seeking women - a challenge for health professionals [105] * Exploring potentials and risks of healthcare interpreting ‘Migrant-Friendly Hospitals’: a European initiative in an age of increasing mobility [106] * Telefondolmetschen - eine Chance zur Überwindung von Sprachbarrieren? [107] Health and ill health of asylum seekers in Switzerland: an epidemiological study [108] * Vermitteln Dolmetscherinnen? Dolmetschen Vermittlerinnen? [109] Pflegende Dolmetschende? Dolmetschende Pflegende? Literaturanalyse [110] * Editorial: Die fremden Sprachen, die fremden Kranken: Dolmetschen im medizinischen Kontext [111] On institutionalising interpreter services within transcultural care Overcoming language barriers with foreign-language speaking patients: a survey to investigate intra-hospital variation in attitudes and practices [112] * Improving patient-provider communication: insights from interpreters [113] * Contextualising cultural competence training of residents: results of a formative research study in Geneva, Switzerland [114] *	Geneva context-related information Foreign languages in hospitals [115] Nur übersetzen? Dolmetschen, vermitteln und schlichten in Gesundheitsinstitutionen [116] Wirkt interkulturelle Mediation integrierend? Materialienband des Projektes NFP51-405140-69224 [26] Intercultural mediation: Does it contribute to inclusion? Comparing policies and practices in the sectors of health, education, social and legal services [117] Telefondolmetschen im Spital [118] Nur übersetzen? Dolmetschen, Vermitteln und schlichten in schweizerischen Gesundheitsinstitutionen [119] Dolmetschen im Spital: Mitarbeitende mit Sprachkompetenzen erfassen, schulen und gezielt einsetzen [120] Interkulturelle Mediation: welche Form der Integration? [121] Dolmetschen, Vermitteln, Schlichten – Integration der Diversität [122] L’accès aux soins des patients allophones [123] Droits du patient migrant: quelles sont les bases légales de la consultation médicale en présence d'un interprète?[124] Diversity and equality of opportunity. Fundamentals for effective action in the microcosm of the health care institution [125] Des ponts linguistiques pour mieux guérir -L’interprétariat communautaire et la santé publique en Suisse [126] Migration et santé - Résumé de la stratégie fédérale phase II (2008 à 2013) [127] The Amsterdam declaration, quoted in Saladin [125] Interkulturelle Vermittlungstätigkeiten in Polizei und Justiz des Kantons Genf [128] La médiation interculturelle dans le système scolaire genevois [129] La médiation interculturelle dans les CASS (Centre d’action sociale et de santé) [130] La médiation interculturelle dans la prison à Genève [131]
2011–2016 (‘equity’)	On mainstreaming and rolling out interpreter services with an equity-based framework, Geneva A ‘migrant-friendly hospital’ initiative in Geneva, Switzerland: evaluation of the effects on staff knowledge and practices [132] * The ‘migrants patients reference nurse’: an institutional response to improve the care of vulnerable patients in a university hospital [133] Quality in practice: integrating routine collection of patient language data into hospital practice [134] * Staying in the middle: A qualitative study of health care interpreters’ perceptions of their work Interpreting. [135] * The relevance of clinical ethnography: reflections on 10 years of a cultural consultation service [136] *	Geneva context-related information Inequalities in Health Care for Migrants and Ethnic Minorities [137] Do language barriers increase inequalities? Do interpreters decrease inequalities? [138] Migrants allophones et système de santé - Enjeux éthiques de l'interprétariat communautaire [139] The health of migrants and refugees [140]

### Data analysis

I analysed the data using qualitative content analysis. ‘Qualitative descriptive studies have as their goal a comprehensive summary of events in the everyday terms of those events.’ ([141]:334). In my approach to qualitative analysis, I found Hsieh’s and Shannon’s (2005) distinction helpful. They differentiate between three approaches: conventional, summative and—the one we chose—directed content analysis: ‘With a directed approach, the analysis starts with a theory or relevant research findings as guidance for initial codes’ ([142]:1277). My analysis is ‘directed’ because I draw on existing research and existing theories (migration and globalisation, diversity, integration, health(care) disparities, equity). To quote the authors again, ‘The main strength of a directed approach to content analysis is that existing theory can be supported and extended’ ([28]:1283), and we would add existing concepts, frameworks and policy developments.

I coded, first, the history of health care interpreting according to features of language barriers and language assistance programmes. This yielded the different phases of the emerging institutionalisation of interpreter services. Second, I developed the themes (or categories), according to the four areas of Hsieh’s Bilingual Health Model [21]: (1) communicative goals: roles, (2) individual agency, (3) system norms and (4) QEC—quality and equality of care. Table 2 summarises the Bilingual Health Model.

**Table 2:** Hsieh’s Bilingual Health Model

Summary of the BHC model with its four areas or circles	
A. **Communicative Goals**: The basis of the BHC Model is that interpreter-mediated medical encounters are seen as a goal-oriented communicative activity. ‘In everyday talk, individuals hold multiple goals (e.g. task, identity and relationship goals) that are often negotiated and coordinated rather than explicitly discussed’ ([15]:137).	
B. **Individual agency**: In the BHC Model, individual agency is the condition needed for the fulfilment of communicative goals. Individual agency is the ‘socially constructed and contextually situated self that is rooted in everyday practices and sites that call forth and supply its meanings’ ([15]:139).	
C. **System norms** ‘move the understanding of interpreter-mediated interactions beyond the examination of individual performances and behaviours. Each individual in an interpreter-mediated medical encounter assumes certain roles, functions and behaviours under the influences and frames of the system(s)’. System means ‘the social systems and cultures in which there are specific norms, values and worldviews that are imposed upon individuals within the system’ ([15]:140). It appears that providers belong to the ‘culture of medicine’, in which there are specific views about conceptualising health and illness: this, in turn, can make a patient’s cultural representations ‘incompatible if not incomprehensible’.	
D. **Quality and Equality of Care (QEC)**: QEC is the overarching value of the BHC Model. ‘Although QEC can be a communicative goal when applied in context, it also serves as an all-encompassing value that integrates differences between systems, providing an ultimate value that guides the interpretation of competing systems. In other words, when individuals experience conflict due to competing or conflicting system norms, they rely on the guiding value of QEC to resolve their differences ([15]:143).	

### Author’s stance

The author has been involved in different roles at GUH over many years. With his background in public health, epidemiology, nursing and nursing research, he participated at different states in setting up and coordinating the implementation of health care interpreting in Geneva. Also, he has both an insider view (employee at GUH), as well as an outsider view, due to research and clinical involvement in other contexts as well (including international health research and global health projects). Therefore, the study reflects a single viewpoint. I have applied an auto-ethnographic approach to describe how healthcare interpreting evolved through different phases. Auto-ethnography is one of the newer developments in ethnographic inquiry, in which the researchers’ own thoughts and perspectives form a central element of a study [143].

### Study setting

In Geneva, Switzerland, 43.5% of the population is foreign-born [144, 145], and about half of the foreigners speak a language other than French as their primary language ([32]:438). Geneva, with its multicultural and multilingual society, is the Swiss city with the highest number of foreigners and migrants. The public health services in Geneva, all of them under the structure of the *Hôpitaux Universitaires de Genève*, cater for even higher proportions of foreigners and migrants. Already in 1992, foreign patients from 131 nationalities attended the medical outpatient clinic; they made up 56% of the total of 14,600 consultations [58]. While this is the one service (‘policlinique’, meaning clinic for the polis, the city) that always had the highest number of non-Swiss patients, other departments account for proportions of migrants, as high as 40% up to 50% [112].

Geneva University Hospitals (GUH) is a 2000-bed, public hospital group, organised into 11 medical departments, each containing 2 or more clinical services. The 11 departments include Anesthesiology/Pharmacology/Intensive Care, Surgery, Child and Adolescent Health, Gynecology and Obstetrics, Community Medicine and Primary Care, Genetic Medicine and Laboratory, Internal Medicine, Clinical Neurosciences, Psychiatry, Rehabilitation and Geriatrics, and Imagery and Information Sciences [112].

At the GUH, the term most used to describe foreign-language speakers (and patients) is ‘allophone’, a term initially derived from linguistics, and it means patients whose first language is not the one spoken in the country they live in ([146]:205).

### Phases in the evolution of healthcare interpreting

In the following section, I describe the different phases in the institutionalisation of healthcare interpreting and their characteristics as they emerged in the case study, and, second, an analysis by applying the BHC model against the experience at the Geneva University Hospitals.

#### Phase 1: service initiation-patchy appearance of healthcare interpreting

In the early 1990s, interpreters were occasionally used at GUH at first, in a small service that cared for refugees and asylum seekers, and, later on, in the community medicine department with its outpatient services.

The linguistic landscape at that time was that around 8% of residents in Switzerland did not speak a Swiss language, German, French or Italian. Among these, the main languages were Bosnian-Croatian-Serbian, Portuguese, Turkish, English and Albanian [147].

Until the 1980s, labour migration largely dominated the interaction between host society and migration, this changed in the 1980s [41]. At this time, the age of asylum seekers and refugees began, that is people from countries in war or political unrest. Also, these new arrivals did not come from neighbouring countries (Italians in the 50s and 60s) and Mediterranean countries (Portuguese and Spanish in the 70s) or Turkey (60s, 70s), but from unknown countries where unknown languages were spoken (e.g. Sri Lanka).

Before this, language policies were relatively easy; those coming from the outside of Switzerland needed to learn a Swiss language (that made it easy for Italians; they never were told to learn German or French). Assimilation was the only term for integration politics [148]. That meant that interpreter services were unthinkable. Unlike for example in Australia, where immigrants were actively invited after World War II, to work and to stay [18] and where interpreter services were provided so as to give incentives.

Another element increased this delay in reacting to the new language needs: there has been a *bricolage linguistique* within Swiss language communities, a muddle through approach meaning the French-speaking Swiss just try to use a bit of their German knowledge they had from school, which was often of poor quality. The same applies to German-speaking Swiss having difficulty in basic communication with French-speakers; a bit less the Italian Swiss: being a minority, they always had to learn at least one or two other Swiss languages (German, French).

Against this backdrop of languages spoken (or not spoken), it is evident that something had to be done. Since nothing came from public providers, it was an NGO that stepped in. In 1993, the Geneva Red Cross, in close partnership with the Community Medicine Department at GUH, set up an interpreter service providing interpreters in 43 languages for foreign-language speakers.

This phase, spanning 1992 to 1995, can be summarised this way: Healthcare interpreting is emerging in Geneva. There has been a need for interpreters in languages including Tamil, Kurdish, Turkish, BCS (Bosnian, Croatian and Serbian, or ‘South-Slavic’), Albanian, Arabic and Portuguese (for Angolan refugees). Qualified interpreters were provided by an NGO. Only very few clinics work with interpreters (outpatient department being the main user, and gynaecology/obstetrics, psychiatry and paediatrics) occasionally using interpreters. No formal agreement or convention between NGO and hospitals existed so far. Apart from what we call qualified interpreters , a variety of interpreters are used, ranging from cleaning staff, health professionals, volunteers (from embassies for example), patient relatives and friends.

#### Second phase: growth and formalisation—attempts to formalise healthcare interpreting services

In the next phase, it becomes clear that there is an urgent need to formalise healthcare interpreting. Since most clients (meaning clients needing interpretation) come from countries in war and are refugees, the need for skilled interpreter becomes equally urgent. Refugees and asylum seekers concentrate on one singular unit (*Unité de médecine des voyageurs et des migrations*, UVM) and its overall department (*Médecine Communautaire*). It is here that most interpreters are needed. And it is also here that the highest demands are put on interpreter skills. In a representative sample of asylum seekers attending the UVM [5], we found that more than half of the asylum seekers came from Europe, mainly the Balkan regions, and a third of them from Africa; most asylum seekers were men (72%) and that the median age was 26.5 years. Traumatic events prior to migration were described by 63%, and severe physical and psychological symptoms were reported by 19%. Nurses referred 36% of all refugees to further medical care and 6% to psychological care. The study found that the presence of interpreters significantly influenced the detection of symptoms and exposure to traumatic events, as well as the referral to further care, especially mental health care. At the same time, the study provided some evidence suggesting that relatives serving as ad hoc interpreters did not improve psychological screening and were thus not a promising strategy when addressing language barriers. Asylum seekers apparently felt uncomfortable acknowledging psychological suffering in the presence of family members because they wanted either to protect them from painful narratives or avoid some kind of stigmatisation. ‘In conclusion, our results suggest that addressing language barriers by using trained interpreters in primary care centres may improve the detection of traumatised asylum seekers and increase their appropriate referral to mental health care’ ([5]:511).

Still, interpreters were used mainly (almost only) in the Community Medicine Department. However, an unexpected breakthrough in the development of healthcare interpreting in Geneva came with the Kosovo crisis in 1999. There were thousands of Kosovari (and Albanian-speaking) refugees arriving in Geneva. This triggered the Geneva government (*le conseil d’état*) to allocate extra-budgets for public institutions including health, education and social institutions, so that these could use and pay Albanian-speaking interpreters, whenever they had Kosovari patients facing language barriers. That helped to overcome reluctance in calling in an interpreter not only among doctors and nurses (and other health professionals) but also managers of other medical departments that were always about their finances and having now a chance not to worry. This involved creating a pool of eight Albanian-speaking interpreters. Also, the position of coordinator of interpreter services was created. This person was able to inform all medical departments about the need of healthcare interpreting, to advocate and to encourage the use of interpreters [80].

Summarily, this phase, which lasted from 1995 to 1999, was characterised by the provision of interpreters to asylum seekers, many of them with PTSD (post-traumatic stress disorder). The political events with the ensuing increase of asylum seekers from Kosovo and Albania helped raise awareness in all departments of GUH, including those who were not using interpreters so far.

#### Phase 3: quality - healthcare interpreting as quality of care issue

In the next phase, the process (from a ‘romantic approach’ towards institutionalised forms of healthcare interpreting) can be distinguished when health care interpreting became a quality of care issue. Now healthcare interpreting was no longer ‘nice to have’, or an exotic measure in care provision, or—worse—a kind of paternalistic gesture bestowed to poor people from distant continents. A breakthrough was when in a quality-of-care project there was evidence that interpreters improved the quality of communication with foreign-language speakers. This was done by a ‘before-and-after’ intervention study, in which both patients (allophone and francophone) and physicians completed visit-specific questionnaires assessing the quality of communication. The intervention consisted of training physicians in communicating with allophone patients and working with interpreters [77]. Thanks to this setting with two consecutive samples of patients attending the medical outpatient clinic it was possible to gauge the effectiveness of the training in working with an interpreter, since French-speaking patients served as the control group. The results showed that five out of six of the scores of allophone patients showed statistically significant increases, when compared with French-speaking patients: explanations given by a physician, respectfulness of a physician, communication, overall process of the consultation and information about future care ([77]:541).

Over these years, access to professional interpreters in Geneva has improved gradually thanks to the Geneva Red Cross (GRC), which created an interpreter bank available to Geneva-based social service and health care organisations. GRC interpreters received minimal training (usually four 2-h workshops in which professional standards are communicated) and participate in several supervisory sessions per year. The Geneva University Hospitals established a convention with the GRC, making the GRC interpreters available to all hospital staff needing linguistic assistance. The GRC provides the hospital with a regularly updated list of interpreters, which is accessible to staff via the hospital intranet system. Staff contact interpreters directly to make appointments, and interpreting is paid for by individual hospital departmental budgets [100].

A third event during this phase which helped establish health care interpreting at GUH was the work of the Clinical Ethics Committee that provided guidelines for all departments on the use of interpreters. Interestingly, this was not a group of specialists favouring programmes for migrants, but of experts dealing with ethical dilemmas of all sorts. The ‘avis’ of the CEC was put on the intranet and worked as a stamp of approval for all health professionals to work with interpreters.

Hudelson): ‘While no explicit hospital policy exists that mandates use of professional interpreters, in 2002 the hospital Clinical Ethics Committee took the position that even in the presence of a family member or friend who is well-disposed towards the patient, even if no conflict of interest exists between the patient and the institution that would put a [bilingual] health worker in an awkward position... one should systematically plan on using, at least initially, a mandated, professional interpreter’ ([112]:187).

Summarily, this third phase, spanning from 1999 to 2003 can be characterised as follows: working with healthcare interpreters becomes quality of care issue (and not just ‘le mal nécessaire’ [149] for migrants); second, the service agreement between GUH and the interpreter service, and, third, the avis of the Clinical Ethics Committee recommending the use of professional interpreters.

#### Fourth phase: institutionalisation

In the fourth phase, advances in the following areas helped paving the way towards further institutionalisation: clarifying interpreter roles, costs and coordinated efforts in Switzerland. The questions about who an interpreter is and what her/his role is are recurrent, throughout all phases. During this phase, however, new research shed some light on these issues. In a study into mediation roles in the two Swiss cities of Geneva and Basel, interpreters told how they see their roles [135]. They described four main roles: word-for-word interpreting, intercultural explanation, building patient-provider relationships and accompanying immigrant patients. An additional cross-cutting theme emerged: interpreters facilitating the integration of immigration. Only the first of these is generally regarded as their ‘official’ role. The interpreters take on the additional roles as necessary during a consultation, in response to the needs of the patient and the health professionals. Interpreters who take on the additional roles related to integration have the potential to be important actors in health care services whose patient populations that are increasingly linguistically and culturally diverse.

Another equally recurrent question is that about that interpreter costs. One study helped to clarify the one issue that hospital managers are most keen on knowing: Are migrants really such a burden to the health system [101], and especially the foreign-language speakers [16]? A study was carried out on a representative sample of asylum seekers in Switzerland that investigated the association between language barriers and the costs of health care [138]. We found as expected that asylum seekers showed higher health care costs if there were language barriers between them and the health professionals. Most of these increased costs were attributable to those patients who received interpreter services: they used more health care services and more medical material. However, the study found also and not as expected, these patients also had a lower number of visits to the hospital than patients who faced language barriers but did not receive interpreter services. This study showed that language barriers clearly affect health care costs: interpreter services lead to more targeted health care, concentrating higher health care utilisation into a smaller number of visits. Although the initial costs are higher, it can be posited that the use of interpreter services prevents the escalation of long-term costs.

A third element in this phase of institutionalisation was networking. Coordinated efforts at cantonal, national and international slowly built up and helped establish healthcare interpreting at GUH. In Switzerland, the association interpret was founded. *INTERPRET*, *association suisse pour l'interprétariat communautaire et la médiation interculturelle* is an interesting hybrid organisation which had very disparate stakeholders, interpreters themselves, users of interpreters (mainly from the health field) and institutions (mainly hospitals), and, to make matters even more heterogeneous, with representatives from three language communities in Switzerland. The accreditation of interpreters was initiated. Those advocating for interpreter services (again: mainly in hospitals) had a kind of policy support and were ‘not alone’ in their endeavours and ‘soul mates’. Also, the Swiss Federal Office of Public Health supported (and still supports) that organisation (financially and in terms of service agreements). Concurrently, international networking increased also, which was equally important, on the way to changing GUH to become an institution that is committed to equity.

The main characteristics of this phase (2004–2010) include (i) the clarification of interpreter roles and the link between interpreting and integration, (ii) knowledge generation about interpreter costs and (iii) the networking efforts that enable the GUH to be structurally and ‘ideologically’ embedded in the wider context.

#### Fifth phase: equity

In this last and most recent phase, the focus widened. It is not exclusively about interpreter services, but about intercultural communication, transcultural care, diversity and striving for equity. A 2014 study reports on an intervention at GUH that aimed at increasing cultural competence among health professionals [132]. Working with interpreters was just one of several measures to improve the quality of care for migrants. There were statistically significant differences between practices before and after the training intervention. The broader framework used in this intervention study is, among others, inspired by Migrant Friendly Hospitals’ initiative, an original network and EU project, that teamed up later with the WHO collaborating centre for Migrant-Friendly and Culturally Competent Healthcare (TF MFCCH WHO Europe Task Force). The initiative set itself two main objectives: First, to strengthen the role of hospitals in the EU, and second, to improve hospital services for these groups by defining measures of quality, developing migrant- and minority-friendly routines for service provision and creating migrant and minority-friendly hospital settings [106]. At the same time, GUH is one of the larger partner hospitals of a Swiss-wide Migrant-friendly Hospitals Network that has recently changed its name into H4E, Hospitals for Equity.

The main characteristics of this phase (2011–2016) are the embedment of health care interpreting in migrant-friendly and equity policies on the one hand, and the embedment in cantonal, national and international initiatives on the other.

Summing up this part, the five phases with their main characteristics, main challenges and policy factors are displayed in Table 3.

**Table 3:** From muddling through to institutional approaches to health care interpreting: five phases

Phase	Main characteristics	Main challenges	Political and policy context factors
Phase 1: 1992–1995 Patchy appearance of healthcare interpreting	Emerging interpreter services Languages of asylum seekers Few departments use interpreters Wide array of different interpreter types	No tradition of using interpreters at all What should the profile of a health care interpreter be?	Migration pressure and sharp increase of asylum seekers and refugees ‘to do something’ in terms of language access Up to now policy of assimilation
Phase 2: 1995–1999 First formalised interpreter services for asylum seekers and refugees	Refugees from the Balkan and Africa and Middle-East Many traumatised people Special programme of providing Albanian-speaking interpreters to Kosovo refugees, sensitising all medical departments for interpreting	War, political unrest in countries that make people flee Effect on interpreters interpreting for traumatised people Different services asking for different interpreter services	Migration and mobility as a consequence of globalisation ➔ changing demographics and therefore changing patient population patterns
Phase 3: 1999–2003 Healthcare interpreting provision is an quality of care issue	Research shows, using interpreters can improve quality of care for allophone patients Trainings for interpreters and training health professionals on how to work with interpreters Clinical ethics committee issues advice on the use of interpreters Service agreement with interpreter service	How normative should the hospital be regarding the use of ad hoc vs. professional interpreters? Health professionals use interpreters, and costs increase	Multicultural acceptance increases, multiculturalism instead of assimilation policy Health services become aware that they are to cater for new patient populations
Phase 4: 2004–2010 Towards institutionalised interpreter services	Clarification on different interpreter roles Coordinated efforts at the national level (cantons, other university hospitals) and international level (Migrant-Friendly Hospital initiative Increasingly important role of Interpret’ (the Swiss interpreter association) Costing studies into language barriers appear in Switzerland	Who should fulfil the interpreter roles, and what interpreter roles are called for by health professionals Autonomy of interpreters; they should get organised, they should have their rights addressed	Integration policy instead of assimilation policy Diversity mainstreaming as a health policy approach
Phase 5: 2011–2016 Towards equity	Health care interpreting—a transcultural approach, interventions that target vulnerable groups Interpreting embedded in a package that aims at improving the quality of care of minority groups Hospitals for Equity	A right to have an interpreter? In the area of the epidemic of chronic diseases, there is a need to develop language-accessible chronic disease management programmes	Health care interpreting—an element of global public health? At the same time: resurgence of assimilation politics (‘those migrants just have to learn our language’)

## Results

In this section, I map the BHC model [21] to the Geneva experience and assess how its four circles (communicative goals, individual agency, system norms and quality and equality of care) are dealt with over time (see Table 4). I assessed what happened in the process of institutionalising interpreter services over the last 25 years and examine what could have been done differently to optimise the interpreter services and care.

**Table 4:** Mapping the four circles of the Bilingual Health Communication Model to the Geneva experience

	What happened?	What could have been done?	Suggestions for optimal approach
Communicative goals	Doctors were trained on how to work with interpreters [77]; interpreters were recruited and trained to work in healthcare settings; Learning materials (leaflets, brochures) [150] were prepared, for both health staff and interpreters [98]. In response to increasing arrival of refugees, health care provision for these was adapted; interpreter services offered them the opportunity to communicate their needs [93] Thanks to refugees an awareness to cultural issues among staff was triggered [151] The arrival of high numbers of Albanian immigrants triggered the Geneva government to finance interpreters in all departments [80]	A clear shift from a narrow bio-medical focus in health care towards a culturally sensitive one A clear shift from the paradigm of a conference interpreting towards community interpreting A clear shift from ‘cultural boxification’ (stereotyping) [152] towards a transcultural approach	Three shifts Targeted healthcare for asylum and refugees, that include interpreter service Training Recruitment of interpreters
Individual agency	Interpreters were involved in navigating patients in health facilities People with language skills, communication skills and interpreting skills (especially languages spoken by refugees) were identified and trained to work with refugee patients [58]. The concept of mediation (as opposed to so-called verbatim translation) was introduced [153].	Involve (migrant) patients in the planning of interpreter services A shift from dual communication (provider–patient) towards triadic communication (‘trialogue’) should have been operated in a more systematic way; The fact that the three ‘agents’ in the bilingual interview have competing interests should have been recognised. An explicit shift away from the black box or conduit model should have operated. A shift away from a ‘Swisso-centric’ view on health care should have been operated.	User involvement Expanded interpreter roles (broader scope); while at the same time, interpreter should not become mini-doctors Develop framework where different types of interpreters Develop triadic concept, ‘trialogue’
System norms	The term interpreter has been successfully advocated, instead of the misleading and ‘narrow’ term of the translator [92, 122] Different roles of interpreting were identified and developed [154] Pragmatic approach regarding the question whether only formal interpreters should be used or also informal interpreters [99] There has been progress on language policy; the term allophone is now widely used [126] The use of informal interpreters has not been banned, but the advantages of both basic types (formal interpreters vs. informal interpreters) have been spelt out. [155] Ethical guidelines were elaborated that justified and even required the use of interpreters [139]	The right to health (of migrants) should be clearly spelt out [156] The right to have an interpreter should be warranted and be known to health professionals Insurance companies should have been convinced that interpreter provision is to be reimbursed.	Right to health Right to have an interpreter Ethical committee Integration of bilingual health staff Health insurance covering interpreter expenses
Quality and equality of care (QEC)	The early awareness that there is a need to provide quality of care for migrants as good as for Swiss patients was a decisive factor to provide interpreter services [157] The use of research ‘disguised’ in quality-of-care projects helped to propel the introduction of interpreters [78] Because of the high number of refugees with PTSD, interpreter provision was accelerated [79] Thanks to coordinated national efforts interpreters were certified [82, 158]. H4E, migrant-friendly hospitals provided a framework that allowed introducing healthcare packages tuned towards migrants and refugees; one of them being interpreting [125]	There has been no pro-active policy development regarding health care interpreting Despite research findings showing interpreters’ cost-effectiveness, there has been no acceptance of financing interpreters by administration of hospital departments Inclusion of healthcare interpreting as an essential in programmes, including chronic disease management, health promotion and prevention, patient-centred care and integrated medicine. Telephone interpreting to be rolled out [107] (national efforts (Federal Office of Public Health) Responses to the global pandemic of chronic diseases have so far not addressed language barriers and interpreter support	Focus on quality of care, including research, including monitoring Context-sensitive interpreting (mental health, PTSD) Telephone interpreting Broad framework, whereby health care interpreting package Costing of interpreter services (to show that not using interpreters is too expensive) Comprehensive chronic disease programmes in which interpreters have their place

As a general observation, when applying the BHC Model to the experience on institutionalising interpreter services at GUH, most items of the four circles were implemented, while in the column ‘what could have been done’ fewer items appear.

Features that helped individuals achieve *communicative goals* include doctors’ trainings, interpreters’ trainings, cultural awareness/sensitivity and accommodating health care provision for refugees and asylum seekers. I identified an incomplete shift from a bio-medical focus towards a more comprehensive one that addressed social, cultural and political aspects in care. Also, the risk of cultural stereotyping could not always be avoided, i.e. refugees were classified in culture boxes, and with little competence in having an idea of transcultural communication, that includes also a critical examination of one’s own ‘culture’ [159]. Another change that could have been operated more thoroughly was the shift away from the high ideal of a conference interpreter towards the concept of community interpreting.

In relation to *individual agency*, interpreters were able to become successful communicators that could leverage others’ support to gain more individual agency. They were involved in navigating patients through the health facilities, adopting therefore a wider role than just ‘language transmission’. Learning materials (leaflets, brochures) were prepared, for both health staff and interpreters, on how to become an agent in bilingual health communication; finally, the concept of mediation (as opposed to so-called verbatim translation) was introduced. However, several things could have been done better: migrant patients should have been involved in the planning of interpreter services; a systematic shifting away from dual communication (provider–patient) towards triadic communication (‘trialogue’) could have been implemented, as well as an explicit shift away from the black box or conduit model; and, thirdly, a shift away from a ‘Swiss-centric’ view on health care (I imply with Swiss-centric view that healthcare is for Swiss only and not or only marginally for the non-Swiss) towards a comprehensive transcultural approach; finally, it could have been acknowledged that the three ‘agents’ in the bilingual interview—patient, interpreter, clinician—may have competing interests.

The *system norms* can be regarded as achievements: the term interpreter has been successfully advocated (instead of the misleading and ‘narrow’ term of translator); different roles of interpreting were identified and developed; progress on language policy has been made; the term allophone is now widely used; the use of informal interpreters has not been banned, but the advantages of both basic types (formal interpreters vs. informal interpreters) have been spelt out; and ethical guidelines were put into place, recommending and justifying the use of interpreters. There are three measures that could have been spelt out: the right to health of migrants [156], the right to have an interpreter and the agreement by insurance companies to reimburse interpreter costs.

With respect to *Quality and Equality of Care (QEC)*, the most important achievement was the early awareness that there was a need to provide quality of care for migrants as good as that for Swiss patients; this recognition implied that interpreter provision was integral part of QEC. Because of the high number of allophone refugees with PTSD, interpreter provision was accelerated. Also, the use of research in quality-of-care projects helped to propel the introduction and the rolling out of language assistance programmes. Furthermore, telephone interpreting has started thanks to national efforts (Federal Office of Public Health). The networking with Hospitals for Equity (formerly Migrant-Friendly Hospitals) provided the necessary framework that allowed introducing healthcare packages tuned towards migrants and refugees, one of them being interpreting. What could have been done was to provide pro-active and comprehensive policy development regarding health care interpreting, to provide formal finance and universal access to interpreters irrespective of type of illness or care provision and finally to include language assistance programmes in CDM (chronic disease management).

## Discussion

### Main recommendations

Over the five development phases towards institutionalised healthcare interpreting, the four circles of the BHC model by Hsieh provide the backdrop against which the way from muddle-through interpreting to institutionally grounded health care interpreting can be evaluated.

Can the lessons learnt be applied to other settings? This should be done with caution, because the political and the policy context are likely to be different at each time. Also, when facing another influx of refugees that do not speak the local languages, we cannot afford 25 years to develop the interpreter services. Nevertheless, I propose a list of priority items that might be helpful, when establishing successful bilingual health communication. Main recommendations include the following:

- Primary focus on quality of care. While we framed healthcare interpreting in the 1990s as a quality issue, today I would conceptualise thoroughly any intervention for allophone patients as a quality of care project. From the data gathered by monitoring and measuring, we may derive the logical consequence, i.e. language assistance programmes. With that focus, there is no need to refer to the need of research (health administrators do not want to invest in research), and there is no need to target migrants or asylum seekers, since it is just common sense that ‘care that is not for all is not quality care’ (to paraphrase the US National Quality Forum). Quality-of-care projects imply also costing, an area of healthcare interpreting that is particularly lagging behind. There are a few studies that show that using interpreters is cost-effective [15, 160, 161], and, conversely, that not using interpreters is too expensive when allophone patients are suffering from chronic conditions [16, 102]. Since QEC is the largest construct in the BHC model (the outer circle), you cover a lot when you address quality of care. Moreover, quality care might be the only way to bring insurance companies to reimburse interpreters.
- Heed to the context of war, having in mind that for mental health problems including PTSD, interpreters are indispensable. Do not talk about immigrants, foreigners or strangers in general, but be specific, referring to asylum seekers for example. Also, be specific about language assistance needs for particular groups. This helps avoid triggering prejudice and preconceived opinions. For example, in the US (where a lot of the literature has been accumulated), Hispanics were initially the one group that increased the availability of interpreters [162, 163]. In Switzerland, the need for interpreters arose because of asylum seekers from war-torn countries (Balkan, Middle-East, Africa), many of them with PTSD.
- Another suggestion for the optimal approach is simply: training. Train interpreters and train those working with interpreters: physicians, nurses, physiotherapists etc. Use the whole spectrum of existing training opportunities, from regular, basis training to routine continuing education. Courses on healthcare interpreting can even be run simultaneously, i.e. doctors with interpreters. Contents of training include not only roles of interpretation, the specifics of different healthcare types, the choice of the interpreter and transcultural competence [159, 164] but also the role of culture [97, 165, 166] and the risk of ‘culturalisation’ or the concept of mediation [167–169].
- Setting up healthcare interpreter services should involve policy development. There are a variety of policy frameworks that facilitate the promotion of healthcare interpreting: integration (as opposed to assimilation and multiculturalism) [122, 148, 170], diversity mainstreaming [125], vulnerability and equity [171], and equity being maybe the most promising approach to embed (‘policy-wise’) healthcare interpreting [137, 153, 172–174].
- A slightly different way of framing healthcare interpreting is to recognise that, first, chronic conditions are the global pandemic against which primary health care (and public health) struggle, that, second, CDM is the adequate response to this, and that, third, interpreters, therefore should be part of any chronic disease management. Recent literature illustrates chronic care models that integrate healthcare interpreting; a few examples include cancer CDM [175–178], diabetes CDM [85, 179], hypertension CDM [180, 181] and asthma CDM [182].

Summing up, I postulate that healthcare interpreting can evolve from muddling-through towards institutionalised approaches (anchored in a health service and health system) by addressing quality of care, by focussing on the mental health of asylum seekers and by training of both interpreters and users of interpreters and institutional policy based on equity.

### Methodological considerations, limitations and strengths

This paper has, as other case studies, strengths and weaknesses. While a single person writing this historical case study is a potential source of bias, a strength is its triangulation with data of a huge variety, including qualitative, quantitative and mixed methods, with different viewpoints on the matter of healthcare interpreting, inside and outside the institution (health professionals, interpreters, patients, researchers), as well as appraising both (a) journal articles and (b) other (reports, book chapters, unpublished material). With convenience sampling theoretically, some documents may have been missed. This risk is small as the collection of material dealing with health care interpreting over 25 years was carried out systematically by the researcher and the team surrounding him.

The rigour of this qualitative case study can be determined using three criteria: credibility (‘refers to the value and believability of the findings’ [183]), this was ensured by triangulation that confirm and complete the data; dependability/confirmability (comparable to reliability in quantitative research, refers to ‘how stable the data are’ and to neutrality and accuracy of the data) was achieved by gathering multiple perspectives from various sources (e.g. physicians, nurses, interpreters, social science researchers), with one limitation; however, very little was done to obtain the patients’ perspectives (exception the quality of care study that asked patients to rate the quality of communication and language assistance, [77]; the third criteria is transferability: Houghton et al. recommend thick description as the strategy; ‘To determine transferability, the original context of the research must be adequately described so that judgements can be made. (…). The responsibility of the researcher lies in providing detailed descriptions for the reader to make informed decisions about the transferability of the findings to their specific contexts. (…). Ultimately, the reader can decide whether or not the findings are transferable to another context’ ([183]:16).

## Conclusion

The ‘diversity turn’ challenges today’s health services that strive to cater for allophone populations. Traditional policies arguing that refugees just have to learn local languages have failed to provide equitable access to healthcare. At the same time, interpreter services appear to decrease inequalities.

## Data Availability

Not applicable

## Abbreviations

BHC: Bilingual health communication
CDM: Chronic disease management
GRC: Geneva Red Cross
GUH: Geneva University Hospitals
H4E: Hospitals for Equity
MFCCH: Migrant-Friendly and Culturally Competent Healthcare (TF MFCCH WHO Europe Task Force)
NGO: Non-governmental organisation
PTSD: Post-traumatic stress disorder
QEC: Quality and Equality of Care
UVM: Unité de médecine des voyageurs et des migrations
